# Inhibition of LncRNA-NEAT1 alleviates intestinal epithelial cells (IECs) dysfunction in ulcerative colitis by maintaining the homeostasis of the glucose metabolism through the miR-410-3p-LDHA axis

**DOI:** 10.1080/21655979.2022.2037957

**Published:** 2022-03-28

**Authors:** Siyi Ni, Yingchao Liu, Jihong Zhong, Yan Shen

**Affiliations:** Department of Gastroenterology, The Second Affiliated Hospital of Zhejiang Chinese Medical University, Hangzhou, Zhejiang, China

**Keywords:** lncRNA NEAT1, ulcerative colitis, intestinal epithelial cells dysfunction, glucose metabolism, miR-410-3p

## Abstract

Dysfunction of intestinal epithelial cells (IECs) leads to intestinal epithelial barrier damage and critically involves in the pathogenesis and development of ulcerative colitis (UC). Accumulating studies revealed essential functions of non-coding RNAs in UC. LncRNA NEAT1 (long non-coding RNA nuclear paraspeckle assembly transcript 1) is frequently dysregulated in diverse human diseases. Currently, the precise roles of NEAT1 in the dysfunction of IECs during UC remain unclear. We report NEAT1 was significantly upregulated in IECs from UC patients. In addition, microRNA-410-3p was remarkedly suppressed in IECs from UC patients. Silencing NEAT1 effectively ameliorates the LPS-induced IECs dysfunction. Bioinformatical analysis, RNA pull-down and luciferase assays illustrated that NEAT1 sponged miR-410-3p to downregulate its expression in IECs. Interestingly, the glucose metabolism was obviously elevated in IECs from UC compared with normal colon tissues. Furthermore, NEAT1 promoted and miR-410-3p suppressed glucose metabolism of IECs. We identified lactate dehydrogenase A (LDHA), a glucose metabolism key enzyme, was a direct target of miR-410-3p in IECs. Rescue experiments verified that restoration of miR-410-3p in NEAT1-overexpressing IECs successfully overcame the NEAT1-promoted cell death under LPS treatment by targeting LDHA. In summary, these results unveiled new roles and molecular mechanisms for the NEAT1-mediated IECs dysfunction during the ulcerative colitis.

## Graphical Abstract

**Figure:**
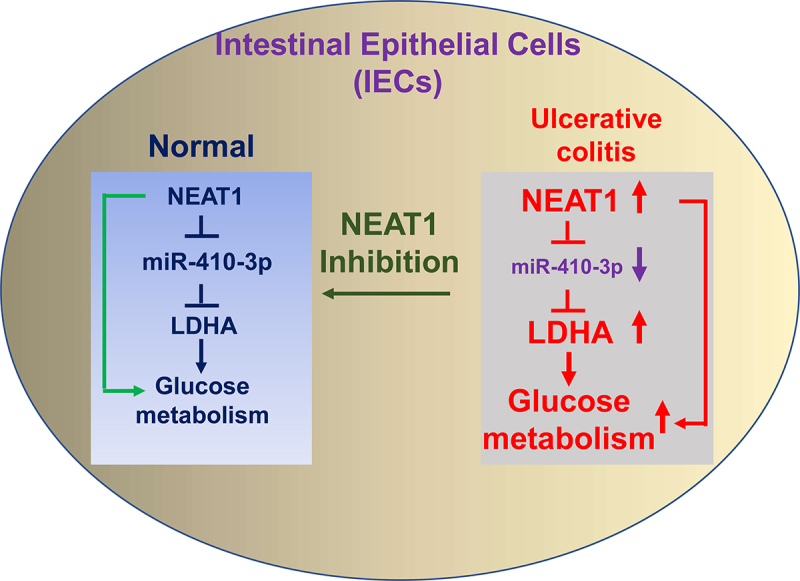

## Introduction

Ulcerative colitis (UC), a gastrointestinal disorder, is one of the two major subtypes of chronic inflammatory bowel disease [1]. UC is characterized by intestinal epithelial injury, such as intestinal epithelial cell death, ulceration, and mucosal erosion [2], leading to a considerably high morbidity rate worldwide. Currently, the precise molecular mechanisms of UC remain unclear. The intestinal epithelial barrier is composed of diverse types of intestinal epithelial cells (IECs) and intact tight junction (TJ) [3]. The barrier functions as a physically and immunologically defensive biolayer against toxins and pathogens [4]. Excessive dysfunction of IECs could alter the physiological characteristics of tight junctions, leading to intestinal epithelial damage [4]. Thus, understanding the molecular mechanisms of dysfunction of IECs in UC will benefit to the treatment of UC patients.

Long non-coding RNAs (lncRNAs) are a group of RNA transcripts with relatively large size (>200 bp in length), and without protein-coding capacity [5]. LncRNAs are known as important mediators for various physiological and pathological processes by modulating target genes expressions [6]. Moreover, aberrant expressions of lncRNAs are tightly associated with various diseases, including UC [7]. Recent study revealed that long non-coding RNA SNHG5 was upregulated in intestinal mucosa tissues of UC patients to regulate ulcerative colitis via microRNA-375/Janus kinase-2 axis [8], suggesting lncRNAs are crucial regulators and biomarkers in UC. LncRNA-NEAT1 (long non-coding RNA nuclear paraspeckle assembly transcript 1) is frequently dysregulated in diverse human diseases [9]. However, the precise roles and molecular mechanisms of NEAT1 in IECs dysfunction during UC remain unclear.

Targeting glucose metabolism has been reported as a new therapeutic strategy against tumors, which display a glucose addictive phenotype similar to that from inflammatory cells [10]. This was proposed by Otto Warburg [11]. Recent studies demonstrated that glucose metabolism was the preferred metabolic pathway in metabolic profiling of patients with ulcerative colitis and Crohn’s disease (CD) [12,13]. Given the fact that glucose is the preferred source of energy in activated inflammatory cells, targeting glucose metabolism might be a novel treatment option for UC or CD patients.

In this study, we hypothesized that blocking the NEAT1-mediated glucose metabolism in IECs from UC patients could reverse the dysfunction of IECs. Thus, we aimed to investigate the molecular mechanisms of NEAT1 in regulating glucose metabolism in IECs. The potential miRNA targets of NEAT1 will be identified and validated. This study will contribute to the development of the lncRNA-based treatment approaches against ulcerative colitis.

## Materials and methods

### Human tissue samples

The study was approved by the Ethics Committee of the Second Affiliated Hospital of Zhejiang Chinese Medical University. A total of 40 patients who were diagnosed with UC and 40 healthy controls were enrolled in this study in the Department of Gastroenterology, the Second Affiliated Hospital of Zhejiang Chinese Medical University during the time period from April 2017 to July 2019. The informed consent was taken from all the patients. UC patients were screened with no other therapy was given before this study and the first diagnosis. Healthy control patients matched the gender and age distributions of the patient group. Collected tissue specimens during surgery were immediately washed with PBS and frozen into liquid nitrogen then transferred to −80°C for subsequent analysis. This study was conducted in accordance with the Declaration of Helsinki (as revised in 2013).

### Isolation of IECs and cell culture

Intestinal epithelial cells were isolated according to previous reports [14]. Briefly, a small piece of colon tissue was dissected and embedded in RPMI 1640 tissue culture medium (10% FCS). Tissues were cut into 5-mm pieces and rinsed with ice-cold PBS followed by incubation with Ca2+- and Mg2+-free PBS containing 2 mM DTT, 5 mM EDTA, and 10% FCS for 0.5 hour at 37°C. The supernatant was filtered through both 70 and 30 μM MACS SmartStrainers (Miltenyi Biotec). IECs from the supernatant were further purified by 40% Percoll (GE Healthcare, USA). IECs were sorted by flowcytometric analysis using Beckman Coulter Gallios Flow Cytometer. Cells were used in passage 3–6. Cells were cultured in RPMI 1640 culture medium (Invitrogen, USA) supplied with 10% FBS (fetal bovine serum) (Invitrogen, USA) with 5% CO_2_ at 37°C. Regular cell culture medium contains 4500 mg/L and low glucose RPMI-1640 medium contains 1000 mg/L glucose.

### Bioinformatics analysis

Predictions of the lncRNA-miRNA and miRNA–mRNA interaction were performed from the starBase of ENCORI http://starbase.sysu.edu.cn/ [15].

### Transfections of miRNA, siRNA and plasmid DNA

Transient transfections were conducted using the Lipofectamine® 2000 Reagent (Thermo Fisher, USA) following the instructions from the kit. Cells were seeded onto six-well plates (3 × 10^5^ per well) for 24 hours. NEAT1 overexpression vector was constructed from GenePharma (Shanghai,

China). NEAT1 siRNA, miR-410-3p and their negative controls were designed and synthesized from GenePharma (Shanghai, China). The sequences used in this study were: siNEAT1: 5′- GGGACAGACAGGGAGAGATG-3′; siRNA negative control: 5′- UGGUACUGGAUCCUACCUUUCCGUA-3′. miR-410-3p mimic: 5′-AAUAUAACACAGAUGGCCUGU-3′. Plasmid DNA was transfected at 1 µg/ml. siRNA or miRNAs was transfected at concentration of 25 nM. Subsequent experiments were performed 48 hours after transfections.

### RNA isolation and quantitative real-time PCR

Total RNAs from IECs were extracted using a TRIzol kit (Thermofisher, USA). Quality and concentrations of RNA samples were determined using Nanodrop 2000 Spectrophotometer (Thermofisher, USA). The PrimeScript RT Master Mix kit (TaKaRa, Shiga, Japan) was used for cDNAs synthesis. Quantitative real-time PCR reactions of lncRNA NEAT1 and mRNAs were performed using SYBR Green qPCR Master Mix (Thermofisher, USA). Quantitative real-time PCR reactions of miRNAs were performed using SYBR premix Ex Taq kit (Takara, Dalian, China). The qRT-PCR primer sequences were as follows: NEAT1 forward, 5′- TGGCTAGCTCAGGGCTTCAG-3′ and reverse, 5′- TCTCCTTGCCAAGCTTCCTTC-3′; β-actin forward, 5′- AGCACAGAGCCTCGCCTT-3′ and reverse, 5′-CATCATCCATGGTGAGCTGG-3′; miR-410-3p forward, 5′-AGTTGTTCACCACCTTCTCCAC-3′ and reverse, 5′-TATCGTTGTACTCCAGTCCAAGTC-3′; U6 forward, 5′-CTCGCTTCGGCAGCACA-3′ and reverse, 5′-AACGCTTCACGAATTTGCGT-3′. β-actin and human U6 were housekeeping controls for lncRNA, mRNA and miRNA, respectively. Relative gene expressions were calculated by 2^−ΔΔCt^ method.

### RNA pull-down

The lncRNA–miRNA interaction was examined by RNA pull-down assay using the Pierce™ Magnetic RNA-Protein Pull-Down Kit (Thermofisher, USA) according to the protocols from the kit. Sense-, antisense-NEAT1 and scramble control RNA probes were *in vitro* synthesized and labeled with biotin. Cell extracts were incubated with the above probe for 2 hours, followed by incubation with streptavidin agarose beads from the kit. The amount of miRNAs from the RNA-binding complexes was determined by qRT-qPCR analysis.

### Luciferase reporter assay

Wild type (WT) or miR-410-3p binding site mutated (Mut) NEAT1 or LDHA 3ʹUTR was amplified and ligated into pGL3 luciferase vectors (Promega). Cells were plated onto 24-well plates for 24 hours followed by transfection by control miRNA or miR-410-3p with WT- or Mut- NEAT1 or LDHA 3ʹUTR in pGL3 luciferase vector using Lipofectamine 2000 (Invitrogen, USA) for 48 hours. Luciferase activities were measured using Dual-Luciferase reporter assay system (Promega, USA) according to the instructions from the kit.

### Glucose metabolism assay

The glucose metabolism rate of IECs was evaluated by glucose uptake and Extracellular acidification rate (ECAR) measured by Glucose Colorimetric Detection Kit (Thermofisher, USA) and the seahorse metabolic analyzer (Agilent, USA) according to the protocols from the kit. The relative glucose uptake and ECAR from experimental groups were normalized to the data from control groups. Experiments were performed in triplicate.

### Cell viability assay

Cell viability of IECs in response to LPS stimulation was determined by MTT (3-(4,5-Dimethylthiazol-2-yl) assay (Sigma-Aldrich, Shanghai, China). Briefly, cells were seeded onto 96-well plate at density of 8 × 10^3^ cells/well for 24 hours. After LPS stimulation, medium was refreshed. MTT reagent (5 mg/mL, 20 µl) was added into well and incubated for 4 hours. Then DMSO (dimethyl sulfoxide, 100 μl) was added. Absorbance was detected at 450 nm using a microplate reader (Bio-Rad, USA). Relative cell viability was calculated experimental groups were normalized to the data from control groups. Experiments were performed in triplicate.

### Colony formation assay

Intestinal epithelial cells isolated from normal and UC tissues were cultured with regular or low glucose medium for 14 days. Colonies were stained with crystal violet (0.05%) for 2 mins at room temperature then washed by PBS. The survival colonies were recorded under brightfield microscopy.

### Cell apoptosis assay

Intestinal epithelial cells (2 × 10^6^ cells/well) were seeded onto six-well plate for 24 h and then exposed to LPS at the indicated concentrations for 24 hours. Cells were washed with PBS and resuspended in binding buffer from the kit. Cells were stained with Annexin V-FITC/propidium iodide from an apoptosis detection kit (Thermofisher, USA.) according to the manufacturer’s protocol. The apoptosis rate of cells was analyzed using an Accuri C6 flow cytometer (BD Biosciences). Experiments were performed in triplicate.

### Western blot

Intestinal epithelial cells were lysed using RIPA lysis buffer (Sigma-Aldrich, Shanghai, China) on ice for 20 min. Cell lysates were centrifuged for 10 min at 12,000 × g at 4°C. Proteins from supernatants were collected and quantified by Bradford method. Equal amount of protein (40 µg) from each sample was separated by 10% SDS-PAGE followed by transferring to PVDF membranes. Membranes were blocked by 5% nonfat milk in PBST at room temperature for 1 h. Membranes were incubated with primary antibodies (1:1000) at 4°C for overnight. After complete rinsing with TBST, membranes were incubated with horseradish peroxidase-conjugated secondary antibody (Invitrogen, USA) at room temperature for 1 h. Proteins from membranes were then visualized using an ECL plus kit (GE Healthcare, USA).

### Statistical analysis

All data are presented as the mean ± SD (standard deviation). Data were analyzed using GraphPad Prism (v7.0, GraphPad Software, Inc. USA). Experiments were repeated at least three times. Comparisons between two group were conducted using the unpaired Student’s t-test. Multiple comparisons were conducted using One-way ANOVA followed by the Newman-Keuls test. P < 0.05 was considered to be statistical significance.

## Results

### NEAT1 and miR-410-3p are reversely expressed in intestinal epithelial cells from ulcerative colitis

**Figure 1.**
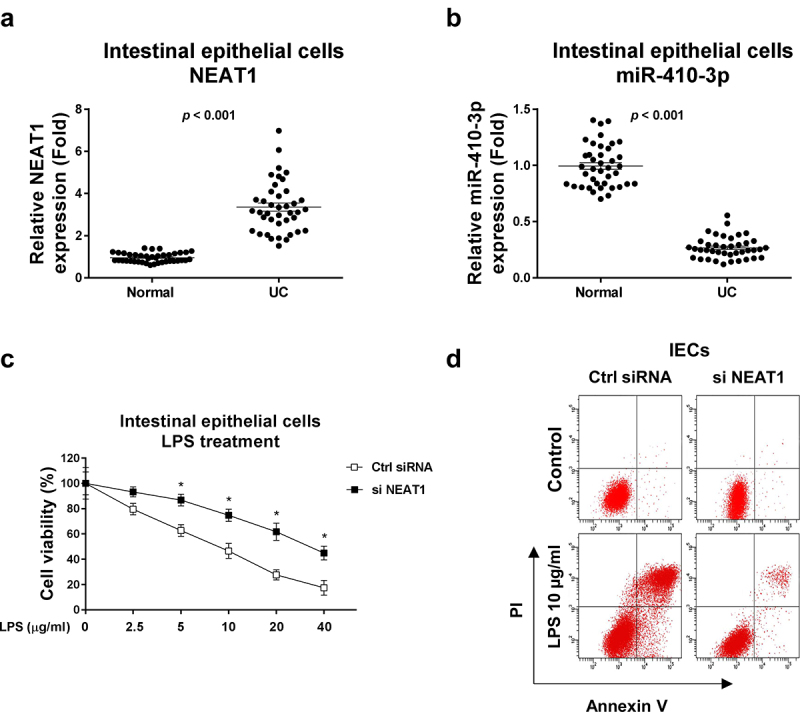
**Expressions of NEAT1 and miR-410-3p in normal and UC**. (a) Expressions of NEAT1 and (b) miR-410-3p were examined by qRT-PCR in normal colon tissues and UC tissues. (c) IECs were transfected with control siRNA or NEAT1 siRNA for forty-eight hours, followed by treatments with LPS at the indicated concentrations for 24 hours. The cell responses were determined by MTT cell viability assay and (d) Annexin V apoptosis assay. *, *p* < 0.05.

This study aimed to investigate the roles and molecular mechanisms of lncRNA NEAT1 and miR-410-3p in the pathological processes of ulcerative colitis. The cellular glucose metabolism of IECs from normal colon tissue and UC will be characterized. The potential miRNA targets of NEAT1 will be identified and validated. Expressions NEAT1 and miR-410-3p were compared in intestinal epithelial cells isolated from ulcerative colitis tissues and healthy controls. Results from qRT-PCR demonstrated that NEAT1 was significantly upregulated and miR-410-3p was remarkedly downregulated in intestinal epithelial cells from UC (Figures 1(a, b)). In summary, the above results suggest NEAT1 is positively associated with UC and miR-410-3p plays reverse roles against NEAT1 in UC.

### Inhibition of NEAT1 ameliorates the LPS-induced IECs dysfunction

Given the crucial roles of IECs dysfunction in UC [4], we assessed whether targeting NEAT1 could contribute to ameliorating IECs dysfunction. Cell viability assay in Figure 1(c) demonstrated that under LPS stimulation, IECs displayed apparent cell death. With relatively low NEAT1 level, the LPS-induced IECs dysfunction was significantly alleviated (Figures 1 (c, d)). These results suggest that NEAT1 could be a potential target for improving IECs conditions during ulcerative colitis.

### NEAT1 sponges miR-410-3p to downregulate its expressions in intestinal epithelial cells

**Figure 2.**
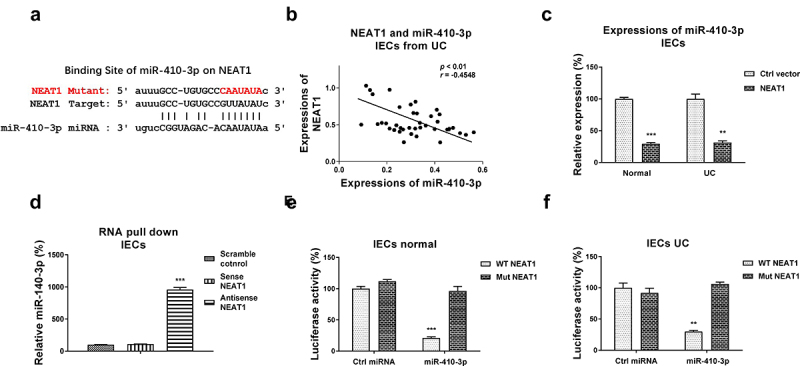
**NEAT1 sponges miR-410-3p in IECs**. (a) Prediction of the NEAT1-miR-410-3p association from starBase. (b) Correlation between NEAT1 and miR-410-3p expression in IECs from 40 UC tissues. (c) IECs were transfected with control or NEAT1 overexpression plasmid, expressions of miR-410-3p were determined by qRT-PCR. (d) RNA pull-down assay was performed in IECs by incubating cell lysates with the biotin-labeled control, sense or antisense NEAT1 probes. Amount of miR-410-3p in the RNA complex was assessed by qRT-PCR. (e) IECs isolated from normal and (f) UC tissues were transfected with WT-NEAT1 or Mut-NEAT1 plus control miRNA or miR-410-3p, luciferase reporter assay was conducted to examine the specific binding of miR-410-3p on NEAT1. **, *p* < 0.01; ***, *p* < 0.001.

We then assessed whether NEAT1 directly regulated miR-410-3p in UC. Previous studies reported that lncRNAs downregulated miRNAs through sponging them to form ceRNA networks, resulting in de-repression of the miRNA-targeted mRNAs expressions [6]. To identify the miRNA targets of the NEAT1 in UC, we firstly searched the potential miRNAs which could supplementarily bind with NEAT1. Among the candidates, miR-410-3p contains putative NEAT1 binding sites (Figure 2(a)). Interestingly, Pearson’s correlation coefficient analysis revealed that expression of NEAT1 was negatively correlated with miR-410-3p in IECs from both UC patients and healthy control colon tissues (Figure 2b, S1A), suggesting NEAT1 inhibits miR-410-3p by direct sponging it in IECs. Intestinal epithelial cells with NEAT1 overexpression showed apparently inhibited miR-410-3p expressions (Figure 2(c)). To test the binding of NEAT1 on miR-410-3p, RNA-pull down assay was conducted. Results demonstrated that only the antisense NEAT1 probe could associate with enriched amount of miR-410-3p, while the scramble control and sense NEAT1 probes could not pull-down significant amount of endogenous miR-410-3p (Figure 2(d)). To validate whether NEAT1 directly binds on the predicted region of miR-410-3p, IECs isolated from normal and UC were co-transfected with luciferase vector containing normal NEAT1 (WT-NEAT1) or binding site mutant NEAT1 (Mut-NEAT1) plus control miRNA or miR-410-3p. Luciferase assay demonstrated that miR-410-3p effectively blocked the luciferase activity of WT-NEAT1 vector (Figures 2 (e,f)). But no significant change of luciferase activity from cells co-transfected with Mut-NEAT1 plus control miRNA or miR-410-3p (Figures 2 (e, f)). Taken together, these results conclude that NEAT1 effectively inhibits miR-410-3p expressions in IECs through sponging it.

### Glucose metabolism is elevated in ulcerative colitis

**Figure 3.**
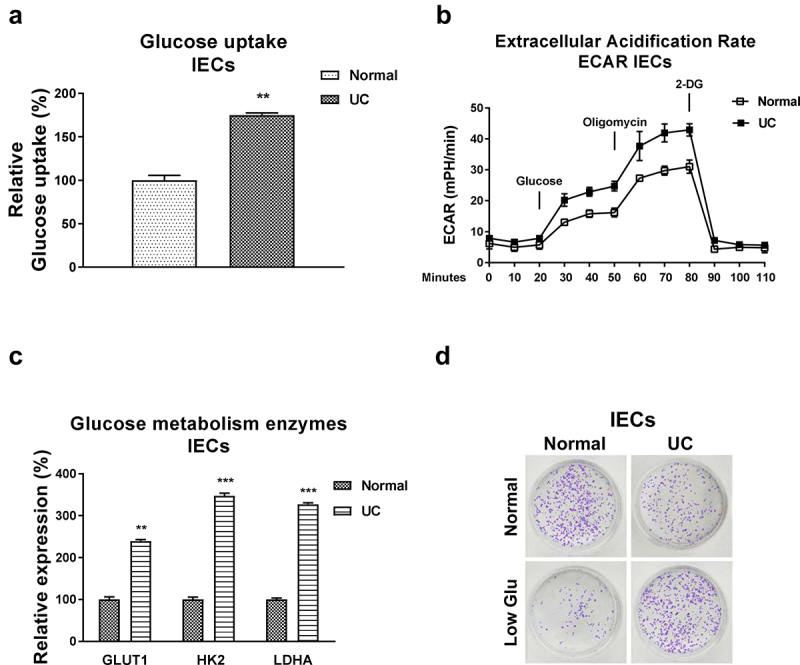
**Glucose metabolism is elevated in IECs from UC**. (a) Glucose uptake and (b) ECAR were detected in IECs from normal and UC tissues. (c) The glucose metabolism key enzymes, Hexokinase 2, LDHA and GLUT1 expressions were detected in IECs from normal and UC tissues by qRT-PCR. (d) IECs from normal and UC tissues were cultured under regular and low glucose conditions, the cell viability was assessed by clonogenic assay. **, *p* < 0.01; ***, *p* < 0.001.

Accumulating studies unveiled that the dysregulated glucose metabolism was tightly correlated with pathological processes of UC [16,17]. We then examined the glucose metabolism rates in intestinal epithelial cells from normal colon tissues and UC patients. As we expected, the glucose uptake and extracellular acidification rate (ECAR), two glucose metabolism key reactions [11], were significantly increased in IECs from UC patients (Figures 3(a, b)). Consistently, expressions of the glucose metabolism key enzymes, Hexokinase 2 (HK2), LDHA, and Glucose transporter 1 (GLUT1) were significantly upregulated in IECs from UC patients (Figure 3(c)). Furthermore, under low glucose supply, IECs from UC showed increased viability compared with that from healthy control cells (Figure 3(d)). Summarily, these results suggest that targeting the upregulated glucose metabolism could benefit the homeostasis of IECs during ulcerative colitis.

### NEAT1 promotes glycolysis of intestinal epithelial cells

**Figure 4.**
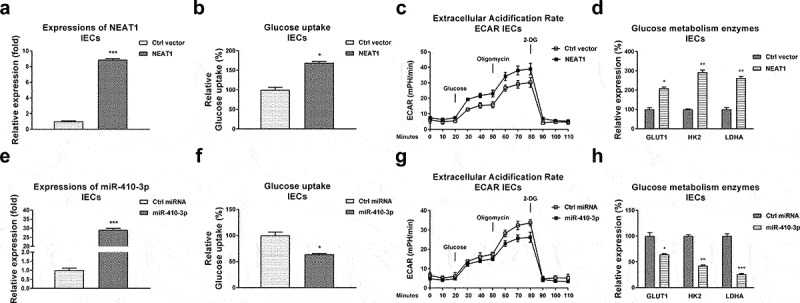
**Reverse roles of NEAT1 and miR-410-3p in regulating glucose metabolism of IECs**. (a) NEAT1 was overexpressed in IECs. (b) Glucose uptake (c) ECAR and (d) glucose metabolism key enzymes expressions from the above transfected cells were examined. (e) Control miRNA or miR-410-3p was transfected into IECs, expressions of miR-410-3p were examined by qRT-PCR. (f) Glucose uptake (g) ECAR and (h) glucose metabolism key enzymes expressions from the above transfected cells were examined. *, *p* < 0.05; **, *p* < 0.01; ***, *p* < 0.001.

Given the above results showed that NEAT1 was upregulated in serum of UC patients, we thus hypothesized that NEAT1 is positively associated with glucose metabolism of IECs. NEAT1 was overexpressed in IECs (Figure 4(a)). Expected results showed that intestinal epithelial cells with higher NEAT1 expressions displayed significantly elevated glucose uptake (Figure 4(b)), ECAR (Figure 4(c)) and glucose metabolism enzymes expressions (Figure 4(d)).

### miR-410-3p suppresses glycolysis of intestinal epithelial cells through direct targeting LDHA

**Figure 5.**
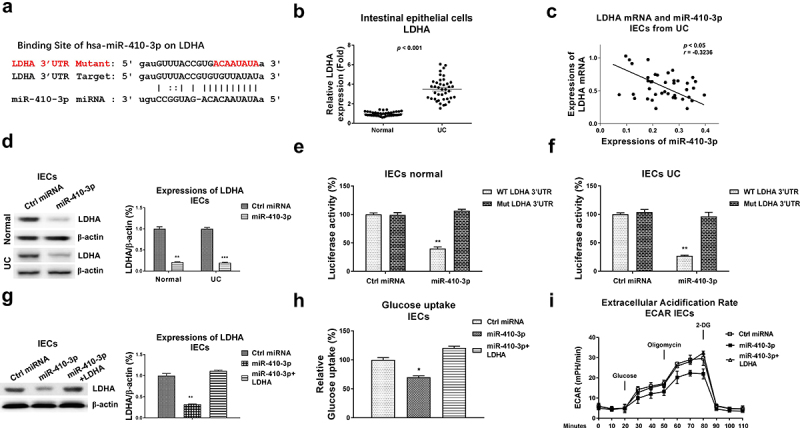
**miR-410-3p targets LDHA to inhibit glucose metabolism**. (a) Prediction of the binding sites of miR-410-3p on 3ʹUTR of LDHA from starBase. (b) Expressions of LDHA were examined in normal and UC tissues. (c) Correlation between miR-410-3p and LDHA in IECs from 40 UC tissues. (d) Control miRNA or miR-410-3p was transfected into IECs from normal and UC tissues. Protein expressions of LDHA were examined by Western blot. (e) IECs from normal and (f) UC tissues were transfected with 3UTR of WT-LDHA or Mut-LDHA plus control miRNA or miR-410-3p, luciferase reporter assay was conducted to examine the specific binding of miR-410-3p on LDHA. (g) IECs were transfected with control miRNA, miR-410-3p or miR-410-3p plus LDHA, expressions of LDHA were determined by Western blot. (h) Glucose uptake and (i) ECAR from the above transfected cells were measured. *, *p* < 0.05; **, *p* < 0.01; ***, *p* < 0.001.

We further evaluated the roles of miR-410-3p in regulating glucose metabolism in IECs. Expectedly, overexpression of miR-410-3p (Figure 4(e)) effectively blocked the glucose uptake (Figure 4(f)), ECAR (Figure 4(g)) and glucose metabolism key enzymes expressions (Figure 4(h)). Accumulating studies revealed that microRNAs regulate target mRNAs transcriptions through binding to the 3ʹUTR of mRNAs. The potential mRNA targets of miR-410-3p were predicted through analyzing the complimentary sequences from non-coding RNA database. Four non-coding RNA service, Targetscan, PITA, miRmap and miRanda consistently predicted that the 3ṣUTR of LDHA, which catalyzes the conversion of pyruvate to lactate in glucose metabolism pathway [11], contains putative-binding sites for miR-410-3p (Figure 5(a)). Clinically, LDHA was detected to be significantly upregulated in IECs from UC compared with health controls (Figure 5(b)). Moreover, Pearson’s correlation coefficient analysis unveiled that expression of LDHA was negatively correlated with miR-410-3p in IECs from both UC tissues and healthy control colon tissues (Figure 5(c), S1B), suggesting LDHA is a potential target of miR-410-3p in IECs during pathological processes of ulcerative colitis. To verify whether miR-410-3p could target LDHA in UC, IEC cells from both health control and UC tissues were transfected with control miRNA or miR-410-3p. Expressions of LDHA were significantly blocked by miR-140-3p in IECs (Figure 5(d)). To validate whether miR-410-3p directly bond to the predicted 3ʹUTR region of LDHA, luciferase reporter assay was performed in IECs from health control and UC tissues (Figure 5(e, f)). Results from Figure 5(e, f) consistently showed miR-410-3p significantly blocked the luciferase activity of vector containing WT-LDHA 3ʹUTR, but not vector containing Mut-LDHA 3ʹUTR. These results demonstrated that miR-410-3p directly targets LDHA in IEC cells.

We then asked whether the miR-410-3p-suppressed glucose metabolism of IECs was through targeting LDHA. Thus, rescue experiments were performed by transfection of control miRNA, miR-410-3p alone or plus LDHA overexpression vector into IECs. Restoration of LDHA in miR-410-3p-overexpressed IECs effectively rescued LDHA expression (Figure 5(g)), glucose uptake (Figure 5(h)) and ECAR (Figure 5(i)). Taken together, the above rescue experiments clearly unveiled that miR-410-3p suppresses glucose metabolism of IECs via direct targeting LDHA.

### NEAT1 modulates the LPS-induced dysfunction of intestinal epithelial cells through the miR-410-3p-LDHA axis

**Figure 6.**
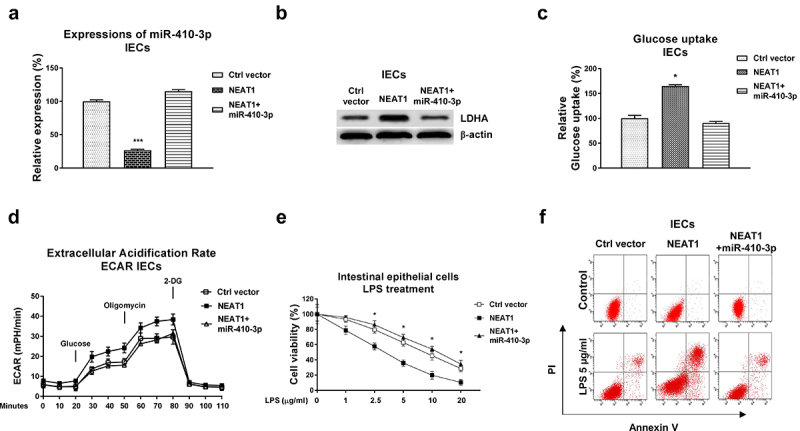
**Roles of the NEAT1-miR-410-3p-LDHA in dysfunction of IECs**. (a) IECs were transfected with control plasmid, NEAT1 alone or plus miR-410-3p, expressions of miR-410-3p and LDHA were determined by qPCR and (b) Western blot. (c) Glucose uptake and (d) ECAR from the above transfected cells were measured. (e) The above transfected cells were treated with LPS at the indicated concentrations. Cell viability and (f) Cell apoptosis were determined by MTT assay and Annexin V apoptosis assay. *, *p* < 0.05; ***, *p* < 0.001.

We investigated whether the NEAT1-mediated glucose metabolism contributed to dysfunction of intestinal epithelial cells through modulating the miR-410-3p-LDHA axis. Consequently, mechanism rescue experiments were conducted by co-transfection of control vector, NEAT1 overexpression plasmid alone or plus miR-410-3p into IECs. Overexpression of NEAT1 significantly blocked miR-410-3p and upregulated LDHA expressions, which were further overcome by miR-410-3p overexpression (Figures 6(a, b)). Co-transfection of NEAT1 plus miR-410-3p successfully recovered the glucose uptake (Figure 6(c)) and ECAR (Figure 6(d)) compared with results from NEAT1 overexpression alone and control cells. Expectedly, overexpression of NEAT1 exacerbated the LPS-induced intestinal epithelial cells death (Figure 6(e, f)). Restoration of miR-410-3p in NEAT1-overexpressed IECs successfully rescued the LPS-induced dysfunction of IECs from cell viability assay (Figure 6(e)) and apoptosis assay (Figure 6(f)). Taken together, these results uncovered that blocking the NEAT1-promoted glucose metabolism benefits IECs from UC through modulating the miR-410-3p-LDHA axis.

## Discussion

Accumulating studies unveiled that dysfunction of intestinal epithelial cells was tightly correlated with the pathogenesis and development of ulcerative colitis [1,2]. However, the molecular mechanisms of the IECs dysfunction during UC are still under investigation. This study aimed to evaluate the roles of non-coding RNA NEAT1 and miR-410-3p in the cellular glucose metabolism and LPS-induced dysfunction in UC, suggesting that targeting the lncRNA-NEAT1-miR-410-3p-LDHA axis could be a potentially therapeutic strategy against UC.

Studies have uncovered that lncRNAs are important regulators in occurrence and development of diverse diseases including UC, suggesting lncRNAs are potentially biomarkers and therapeutic targets for treatment of UC. LncRNA NEAT1 is frequently regulated in various human diseases [9,18]. Moreover, particular lncRNAs have been reported to be associated with intestinal epithelial barrier functions [19,20]. For instance, lncRNA BC012900 was a known mediator of IECs apoptosis [19]. In addition, lncRNA H19 participated in the disruption of tight junction of intestinal epithelial barrier by suppressing expressions of TJ protein and zonula occludin 1 (ZO-1) [20]. Yet, the roles of NEAT1 in UC have not been unveiled. Here, we demonstrated NEAT1 was apparently upregulated in IECs from UC tissues compared with normal colon tissues. Blocking NEAT1 effectively attenuated the LPS-induced IECs death, suggesting NEAT1 was positively associated with UC progression.

We investigated the underlying molecular targets of NEAT1 in UC. Mounting evidence indicated that lncRNAs regulated target miRNAs expressions through sponging them to form ceRNA networks [21]. We demonstrated miRNA-410-3p was a NEAT1-sponged miRNA in IECs. Overexpression of NEAT1 effectively blocked miR-410-3p. Consequently, we identified the glucose metabolism key enzyme, LHDA as a direct mRNA target of miR-410-3p in IECs from luciferase assay and Western blot.

The ‘Warburg effect’ describe that cancer cells continuously rely on glycolysis for their fast proliferative, invasive, and metastatic nature [11]. Similarly, IECs display traits that they adapted and modulated glucose metabolism accordingly [22–24]. Recent study unveiled that glucose metabolism was the preferred metabolic pathway in patients with ulcerative colitis (UC) and Crohn’s disease (CD) compared with non-diseased controls [12,13], assuming that targeting glycolysis pathway might be a novel and effective treatment approach against UC. Our findings revealed the glucose consumption and extracellular acidification rate were remarkedly elevated in IECs from UC tissues. Moreover, IECs-UC cells exhibited an improved cell proliferation under low glucose supply (1000 mg/L) than those from regular cell culture conditions (4500 mg/L), suggesting excessive glucose metabolism may exacerbate the dysfunction of IECs from UC tissues and blocking glucose metabolism pathway could effectively enhance the viabilities of IECs from UC. Rescue experiments demonstrated the NEAT1-mediated IECs dysfunction under LPS stimulation was through modulating the miR-410-3p-LDHA axis. This study still has limits that these *in vitro* results need to be validated by *in vivo* experiments. Although the NEAT1-miR-410-3p association has been reported in other diseases (23, 24), our data present a first insight into roles of the NEAT1-miR-410-3p-LDHA axis in the LPS-induced dysfunction of IECs.

## Conclusion

In summary, we demonstrate that NEAT1 promotes the LPS-induced IECs dysfunction in UC by sponging miR-410-3p to regulate its downstream target, LDHA. This study will contribute to developing novel therapeutic approaches for the treatment of UC.

## Supplementary Material

Supplemental Material
