# Supplementary figures and images for: Inhibition of LncRNA-NEAT1 alleviates intestinal epithelial cells (IECs) dysfunction in ulcerative colitis by maintaining the homeostasis of the glucose metabolism through the miR-410-3p-LDHA axis
Source: Bioengineered. 2022 Mar 28;13(4):8961–71. doi: 10.1080/21655979.2022.2037957 (PMC9161899; doi:10.1080/21655979.2022.2037957)

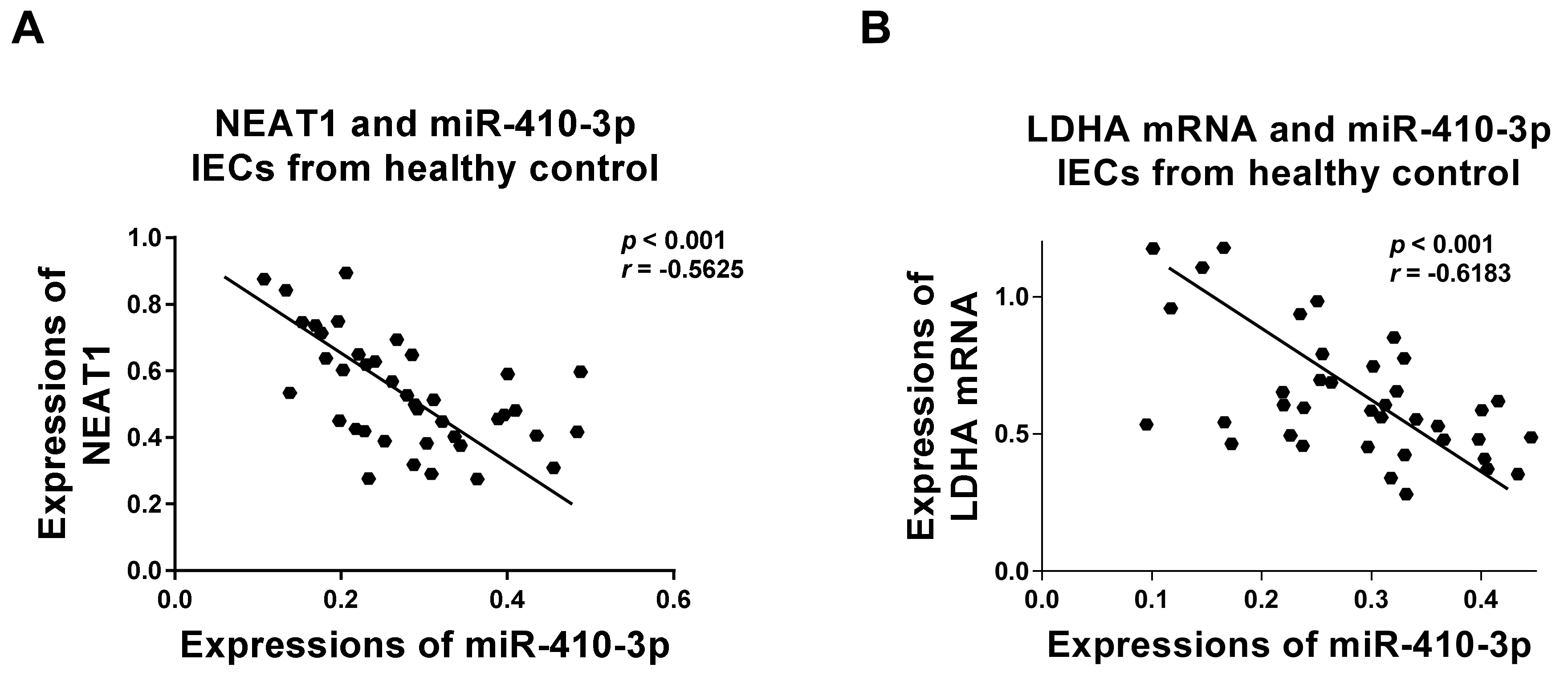

Supplement: Supplemental Material [file KBIE_A_2037957_SM8376.tif]
